# Machine Learning Algorithm for Distinguishing Ductal Carcinoma In Situ from Invasive Breast Cancer

**DOI:** 10.3390/cancers14102437

**Published:** 2022-05-15

**Authors:** Vu Pham Thao Vy, Melissa Min-Szu Yao, Nguyen Quoc Khanh Le, Wing P. Chan

**Affiliations:** 1 International Master Program of Medicine, Taipei Medical University, Taipei 110, Taiwan; m142109002@tmu.edu.tw; 2 Department of Radiology, Thai Nguyen National Hospital, Thai Nguyen 24000, Vietnam; 3 Department of Radiology, Wan Fang Hospital, Taipei Medical University, Taipei 110, Taiwan; wingchan@tmu.edu.tw; 4 Department of Radiology, School of Medicine, College of Medicine, Taipei Medical University, Taipei 110, Taiwan; 5 Professional Master Program in Artificial Intelligence in Medicine, College of Medicine, Taipei Medical University, Taipei 106, Taiwan; khanhlee@tmu.edu.tw; 6 Research Center for Artificial Intelligence in Medicine, Taipei Medical University, Taipei 106, Taiwan

**Keywords:** ductal carcinoma in situ, minimally invasive breast cancer, XGBoost, mammographic, ultrasonographic, breast cancer

## Abstract

**Simple Summary:**

Breast cancer nowadays is the most common cancer among women. Two types refer to whether cancer has spread or not: Non-invasive and invasive breast cancers. Invasive ductal carcinoma is responsible for approximately 80% of all breast cancers, and ductal carcinoma in situ accounts for the majority of the remainder. Early identification of types of breast cancers provides breast cancer patients with more options for less invasive therapy. Our study aimed to develop a machine-learning classification model to differentiate ductal carcinoma in situ and minimally invasive breast cancer using clinical characteristics, mammography findings, ultrasound findings, and histopathology features. Our model showed that the five most important features were calcifications on mammograms, lymph node presence, microcalcifications on histopathology, the shape of the mass on ultrasound, and the orientation of the mass on ultrasound.

**Abstract:**

Purpose: Given that early identification of breast cancer type allows for less-invasive therapies, we aimed to develop a machine learning model to discriminate between ductal carcinoma in situ (DCIS) and minimally invasive breast cancer (MIBC). Methods: In this retrospective study, the health records of 420 women who underwent biopsies between 2010 and 2020 to confirm breast cancer were collected. A trained XGBoost algorithm was used to classify cancers as either DCIS or MIBC using clinical characteristics, mammographic findings, ultrasonographic findings, and histopathological features. Its performance was measured against other methods using area under the receiver operating characteristic curve (AUC), sensitivity, specificity, accuracy, precision, and F1 score. Results: The model was trained using 357 women and tested using 63 women with an overall 420 patients (mean [standard deviation] age, 57.1 [12.0] years). The model performed well when feature importance was determined, reaching an accuracy of 0.84 (95% confidence interval [CI], 0.76–0.91), an AUC of 0.93 (95% CI, 0.87–0.95), a specificity of 0.75 (95% CI, 0.67–0.83), and a sensitivity of 0.91 (95% CI, 0.76–0.94). Conclusion: The XGBoost model, combining clinical, mammographic, ultrasonographic, and histopathologic findings, can be used to discriminate DCIS from MIBC with an accuracy equivalent to that of experienced radiologists, thereby giving patients the widest range of therapeutic options.

## 1. Introduction

Among women, breast cancer is the most common cancer in the world aside from nonmelanoma skin cancers [1]. According to the World Health Organization, 2.3 million women were diagnosed with breast cancer in 2020, leading to 685,000 deaths worldwide. Breast cancers are divided into two types, non-invasive and invasive, based on whether it has spread. Among the non-invasive types, ductal carcinoma in situ (DCIS) is the most common [2], accounting for around 84% of all in situ cancers [3]. Pathologists further divide DCIS into four forms: papillary, cribriform, solid, and comedo [4]. High-grade DCIS (comedo) is the quickest to progress to an invasive form. The typical treatment for DCIS has been mastectomy; however, breast conservation therapies for invasive breast cancers have gained acceptance, and initial attempts at using breast-conserving surgeries for DCIS are potentially acceptable [3].

Breast cancers become invasive when they grow outside of the ducts or lobules into the adjacent breast tissue. Up to 70% of these cases have been identified as invasive ductal cancer, also known as infiltrating ductal carcinoma [5], wherein smaller tumor size is associated with a greater survival rate. According to Tabár et al. [6], women with invasive tumors no larger than 14 mm in size and accompanied by casting-type calcifications had a 20-year survival rate of 55%. Mammography screening and improved therapies have increased long-term survival prognoses for patients with invasive carcinomas in this size range [7]. With early identification of cancer type, a greater range of options for those seeking less-invasive therapies becomes available. Many studies have shown the importance of traditional histopathological characteristics in the prediction of breast cancer, such as lymph node status, tumor size, histological grade, margin width, and several biological indicators [8,9,10]. These prognostic factors are appealing in principle and effective with large tumors, but they present challenges when tumors are small [11].

Machine learning (ML) is a part of artificial intelligence that focuses on developing computer algorithms that can change when new information is added. Several studies have shown that ML techniques can be used to quickly diagnose breast cancer with great accuracy [12,13]. However, to our knowledge, no studies have shown that ML algorithms can be used to discriminate between DCIS and minimally invasive breast cancer (MIBC). This study was therefore designed to develop an ML classification model to differentiate DCIS from MIBC using clinical characteristics, mammographic and ultrasonographic findings, and histopathologic features. A successful model can support radiologists in disease diagnosis and decrease the time required to do so.

## 2. Materials and Methods

The Taipei Medical University Joint Institution Review Board approved this study (TMU-JIRB No. N202203003), and patient informed consent was waived due to its retrospective nature.

### 2.1. Study Design

**Figure 1:**
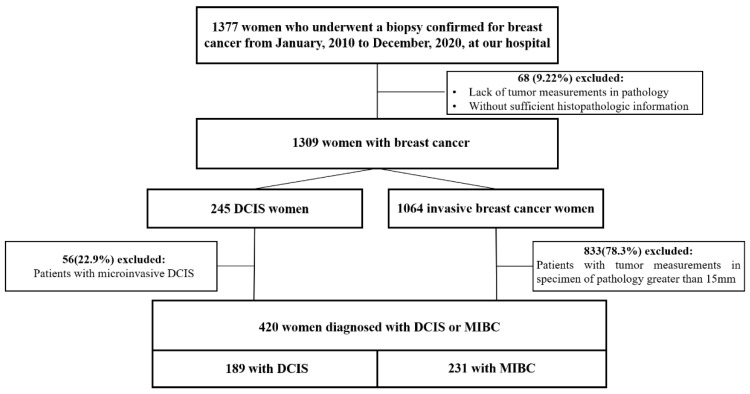
Flowchart of the study population. DCIS: ductal carcinoma in situ; MIBC: minimally invasive breast cancer.

We defined DCIS as discrete spaces filled with malignant cells, frequently surrounded by a recognizable basal cell layer containing normal myoepithelial cells [4]. We defined MIBC as invasive breast cancer found to be less than or equal to 15 mm in size when assessed histologically. Patients who underwent biopsies to confirm breast cancer between 1 January 2010 and 31 December 2020 at Wanfang Hospital of Taipei Medical University were considered eligible for this study (*n* = 1377), leading to electronic medical records reviews. Those lacking tumor measurements in pathology or with insufficient histopathologic information (*n* = 68) were excluded, leaving 1309 patients to be consecutively enrolled in the study. Of these, 245 were found to have DCIS, and 1064 were found to have invasive breast cancer. From the DCIS group, 56 patients were excluded due to microinvasion, as shown via pathology. From the MIBC group, 833 patients were excluded due to excess tumor size (>15 mm). Finally, 420 women with either pure DCIS (*n* = 189) or MIBC (*n* = 231) were included in the study (Figure 1).

### 2.2. Data Acquisition

Our final patient set was split into three non-overlapping sets: 70% (294 patients) for training, 15% (63) for validation, and 15% (63) for testing. Medical records were reviewed to retrieve clinical data, such as age, body mass index (BMI), menopausal status, age at menarche, age at first live birth, family history of breast cancer, use of hormone replacement therapy, and clinical signs of cancer (palpable vs not palpable).

Sonographic features were interpreted using BI-RADS criteria (5th ed.) [14], specifically, breast composition and the breast mass features tumor size, shape, orientation, margin, echo pattern, posterior features, calcifications, vascularity, and elasticity assessment. Architectural distortion, ductal changes, and the status of the axillary lymph nodes were noted. Interval changes on follow-up ultrasound (US) examinations were recorded as well. The sonographic features used in the models are provided in detail in Appendix A (Table A1).

Mammographic findings were also interpreted using BI-RADS criteria [14], specifically those of the masses (size, shape, density, and margin), calcifications (morphology and distribution), and architectural distortion. Asymmetries in density and morphology were also recorded. Interval changes on follow-up mammograms (MMGs) were also reviewed. The MMG features used in the models are provided in detail in Appendix A (Table A1).

Histopathologic findings from excisional biopsies or mastectomy specimens were used as gold standards. The histologic parameters recorded were nuclear grade, presence of comedo necrosis, architectural pattern, and the expressions of ER, PR, and HER-2 (Table A1).

### 2.3. Model Development

**Figure 2:**
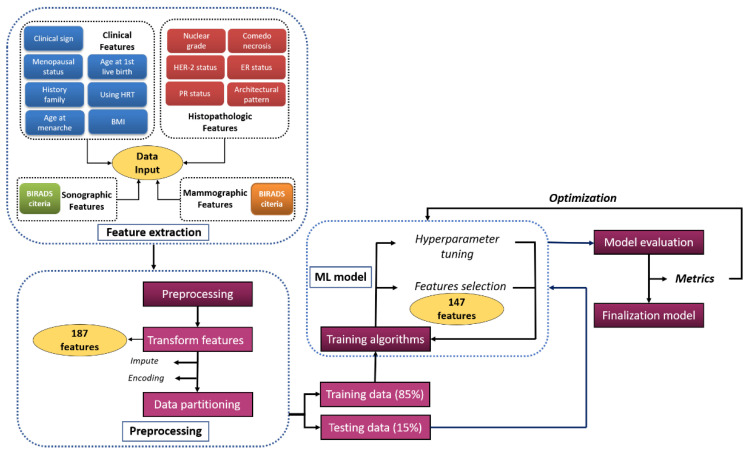
Workflow for the machine learning (ML) model used to distinguish ductal carcinoma in situ from minimally invasive breast cancer.

The process used to develop the classification model is shown in Figure 2. Preprocessing improves the quality of a dataset, supplying clean data that can be used for modeling [15]. In this study, we processed missing values, selected correlation-based features, and labelled features using One Hot Encoder.

Data selection allows the fittest features to be chosen after ranking them using a training dataset. Feature selection, on the other hand, is choosing the combination of features important for classification in preference to those that are less important. The feature selection techniques used in this study were recursive feature elimination methods and application of the XGBoost [16] ‘Feature Importance Scores,’ applying SHapley Additive exPlanations (SHAP) [17] methods.

To develop our model, we used a gradient boosting-based decision-tree-based ensemble ML algorithm in XGBoost in which the computational complexity of determining the optimal split, typically the most time-consuming element of decision tree building methods, is reduced. To find the most important features, multiple values for k were tested using the Select K Best algorithm. We trained the model based on selected features’ importance from the training dataset and wrapped it in the SelecFromModel algorithm. After fitting it with input data, this algorithm extracts the most viable features based on the importance of model weights. Then, we selected the features to be used with the testing dataset to evaluate the model.

Using the same set of features, our model’s performance was compared against other ML algorithms, such as random forest, single vector machine, Gaussian naive Bayes, K-nearest neighbor, and decision tree classifier. The hyperparameters of the XGBoost model were manually tuned and fixed throughout the training process by comparing errors during training, validating using the testing dataset, and automating the determination of the best-fit hyperparameters using a grid search method. To generate more robust models and avoid overfitting, k-fold cross validation was applied when using XGBoost. The hyperparameters of our model are provided in the Table A2 (Appendix B).

We also compared model performance (i.e., classifying DCIS vs MIBC) with the diagnostic performance of radiologists. The testing set of patients was separated into three groups: those to be used with MMG alone, those to be used with US alone, and those where both were used. We also compared the sensitivities and specificities of the groups that comprised the entire sample. Each was independently assessed by 2 radiologists of Wanfang hospital (Radiologist 1 was a first-year resident, and Radiologist 2 had more than ten years of breast imaging experience) for diagnoses. All diagnoses by radiologists and by the model were compared with pathological results. Model performance was evaluated using a number of metrics: accuracy, area under the receiver operating characteristic curve (AUC), precision, recall, sensitivity, and specificity.

### 2.4. Statistical Analysis

Statistical analyses were performed using SPSS, version 25 (SPSS Inc., Chicago, IL, USA). Clinical characteristics of the two types of cancers were compared using the χ^2^ test for categorical variables and the Mann–Whitney test for continuous variables. The McNemar test for sensitivity and specificity was used to compare the diagnostic performance of the model with those of the radiologists. The significance of the differences between evaluation metrics was estimated using the 95% confidence interval (CI), using *p* < 0.05 to find significant differences.

The evaluation metrics were calculated as follows:

- Precision = $\frac{\mathrm{TP}}{\mathrm{TP}+\mathrm{FP}}$
- Recall = $\frac{\mathrm{TP}}{\mathrm{TP}+\mathrm{FN}}$
- Accuracy = $\frac{\mathrm{TP}+\mathrm{TN}}{\mathrm{TP}+\mathrm{FP}+\mathrm{TN}+\mathrm{FN}}$
- F1 score = $\frac{2\;x\;\mathrm{Precision}\;x\;\mathrm{Recall}}{\mathrm{Precision}+\mathrm{Recall}}$
- Specificity = $\frac{\mathrm{TN}}{\mathrm{TN}+\mathrm{FP}}$
- Sensitivity = $\frac{\mathrm{TP}}{\mathrm{TP}+\mathrm{FN}}$

where TP, FP, TN, and FN are true positive, false positive, true negative, and false negative, respectively.

## 3. Results

### 3.1. Study Population

The clinical characteristics of our study group, both as a whole and as divided by cancer type, are summarized in Table 1. The characteristics of the two groups are similar, showing statistically significant differences only in age at first live birth, family history of breast cancer, and BMI group (*p* < 0.05). A larger portion of the DCIS group bore their first child between the ages of 20 and 29 years compared to the MIBC group. Conversely, a larger portion of the MIBC group were nulliparous. The DCIS group had a greater frequency of familial breast cancer history (*p* < 0.05) and a lower frequency of low BMIs (<18.5 kg/m^2^). The MIBC group had the greatest frequency of BMIs in the 24–27 kg/m^2^ range (*p* < 0.05).

As shown in Table 2, the training set consisted of 294 patients (mean [standard deviation] age, 56.8 [11.5] years; BMI, 24.0 [4.6] kg/m^2^) of which 130 (44%) were diagnosed with DCIS. On the other hand, the testing set contained 35 (55.5%) patients with DCIS.

### 3.2. Model Development

#### 3.2.1. Missing Value Processing

**Figure 3:**
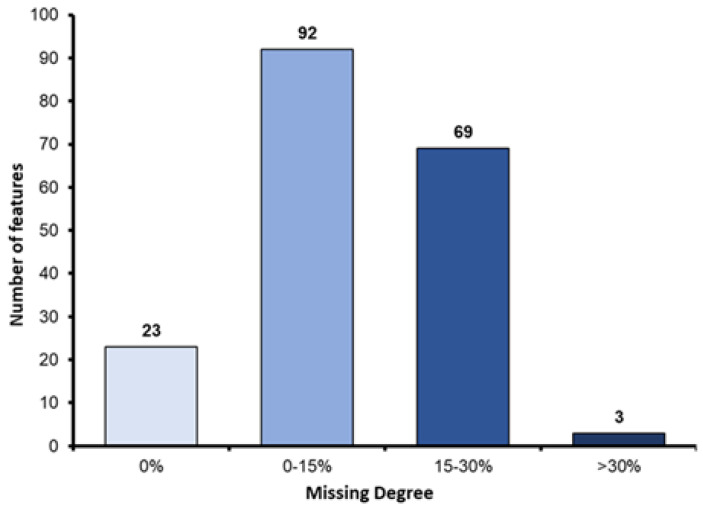
Initial degree of missing features.

We examined 187 features across sonographic, mammographic, and histopathologic findings. Of the 420 patients in our study group, MMG and US were not performed in 99 (24%) and 22 (5%) of the patients, respectively, leading to a number of missing features in those patients. The degree to which these initial features were missing is shown in Figure 3. Those features that were missing more than 30% of the time were excluded from the model. Those features that were missing less than 30% of the time were imputed when missing.

#### 3.2.2. Correlation-Based Feature Selection

**Figure 4:**
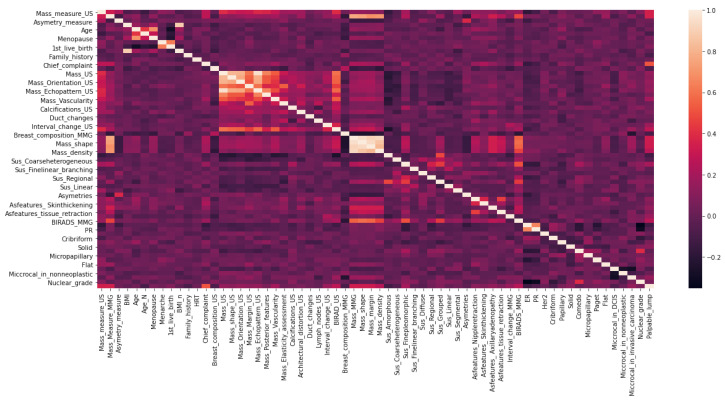
Correlation analysis.

After imputing the missing values, a correlation analysis of the features was performed to avoid using features that were highly correlated with the other features, creating a linear correlation and having little additional impact on the dependent variable. When the correlation exceeded 0.8, that feature was eliminated from the dataset in favor of those with lower means. The correlation analysis is visualized in Figure 4.

### 3.3. Performance of XGBoost and Feature Importance Analysis

**Figure 5:**
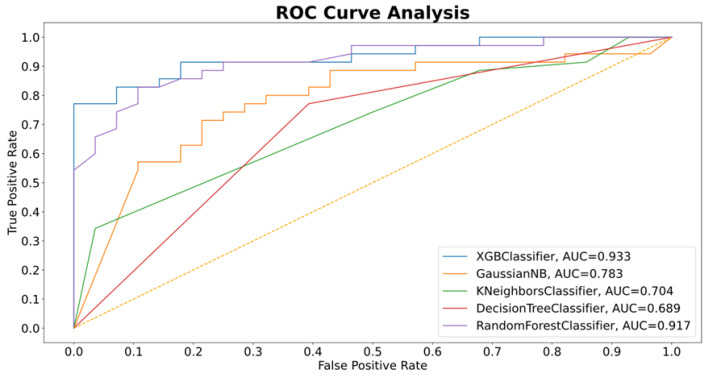
Performances of five models based on area under the receiver operating characteristic curve (AUC).

**Figure 6:**
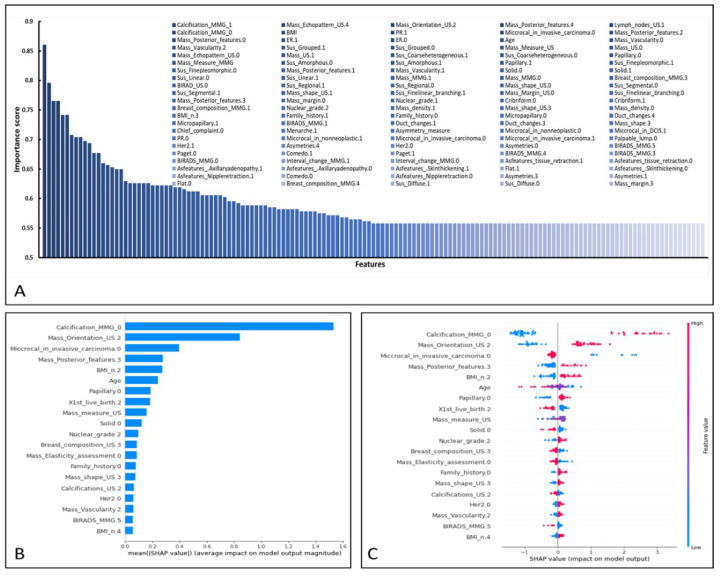
(**A**) Feature importance according to XGBoost. (**B**,**C**) Contribution of the top 20 features as ranked by SHapley Additive exPlanations (SHAP). The features are arranged in descending order on the *y*-axis according to their mean absolute influence on classification. Each dot represents the SHAP value for a certain feature for a certain patient. The SHAP algorithm evaluates all conceivable combinations of features, including and excluding a given feature to evaluate its contribution to the prediction. The farther away from the *y*-axis (positive or negative *x*) a dot is placed, the more impact this attribute has on the machine learning model output for that woman. Dot color indicates the feature’s original value from low (blue) to high (magenta), as indicated by the color array stripe on the right. The color was determined separately for each feature based on the patient’s feature values.

Using the final feature set, the final data were entered into XGBoost, yielding a model accuracy of 0.79 (95% CI, 0.72–0.83) and an AUC of 0.81 (95% CI, 0.73–0.84) as a baseline. To improve on that, a feature importance analysis was used to rank feature importance based on their scores as determined by the XGBoost classifier combined with Select K Best. A set of 147 features (k = 147) yielded the greatest accuracy, F1 score, recall, and precision. As shown in Figure 5, The AUC of the testing dataset reached 0.93 for the breast cancer classification task, producing an overall accuracy of 0.84. Model sensitivity was 0.91 (95% CI, 0.76–0.94) and specificity was 0.75 (95% CI, 0.67–0.83). The scores of the 147 features, based on the XGBoost model, are shown in Figure 6.

By using the SHAP method, we found the 20 most important features that had the most influence on positive prediction of MIBC (Figure 6). See Appendix B (Table A3 and Table A4) for the feature contribution analysis corresponding to this figure.

### 3.4. Performance as Compared with Other ML Methods

The performance of this classification model, using XGBoost, was compared with those of four other algorithms, using the F1 score, recall score, accuracy, precision, and AUC. Results are shown in Table 3 and Figure 5. All five methods showed good results in F1 score, accuracy, and recall; however, XGBoost and the random forest classifier models performed the best using these metrics.

### 3.5. Performance as Compared with Radiologists

Classification by radiologist, when performed using MMG alone or using US alone, yielded lower sensitivity and specificity compared to the use of MMG plus US are shown in Table 4. Compared to the model, Radiologist 1 achieved significantly lower performance metrics for classifying DCIS vs MIBC (*p* < 0.05), achieving a specificity of 0.64 (95% CI, 0.57–0.66) and a sensitivity of 0.74 (95% CI, 0.68–0.79). On the other hand, Radiologist 2 achieved sensitivity and specificity metrics similar to those of the model (*p* > 0.05).

## 4. Discussion

The ML model used clinical features, mammographic features, ultrasonographic features, and histopathologic features extracted from patient medical records to classify DCIS and MIBC. It achieved an AUC of 0.93 (95% CI, 0.87–0.95), a sensitivity of 0.91, and a specificity of 0.75.

Our results support those of others who showed that the age at which a woman bears her first child, a family history of breast cancer, and BMI were associated with the ability to distinguish DCIS from MIBC. Louise et al. reported significant trends in identifying invasive cancer based on the age at which a woman’s first child is born, showing relative risks on the order of 2.2 to 2.7 when the first child is born after 30 years of age compared to before 20 years of age [15]. They further found that a woman’s BMI was slightly associated with the risk of small invasive lesions [15]. The key contributors for classifying DCIS vs. MIBC, therefore, might be age at first live birth, family history of breast cancer, and BMI.

The ML model developed in this study used clinical features, MMG features, US features, and histopathological features to distinguish DCIS from MIBC. When interpreting US and MMG features, BI-RADS criteria (5th ed.) were used. Using all the features, the XGBoost model achieved an AUC of 0.81 (95% CI, 0.73–0.83). By ranking the features and selecting a subset of 147 features based on k score, the model was trained. The five features that contributed the most to the classification task were identified by SHAP analysis as: the appearance of calcification on the MMG, a non-parallel orientation of the mass on US imaging, the presence of microcalcification as identified by histopathology, enhancement of the posterior feature of the mass on US imaging, and BMI group. On the other hand, XGBoost identified the five most important features as: the appearance of calcification on the MMG, the existence of lymph nodes, the presence of microcalcification as identified by histopathology, an irregular shape of the mass on US imaging, and a non-parallel orientation of mass on US imaging. These findings are consistent with those of others. Compared to DCIS, invasive tumors are more irregular in shape, non-parallel in orientation, and yield a hypoechoic or complicated echo pattern [18]. Chen et al. [19] reported that an internal echo pattern was the most important feature differentiating invasive cancers from DCIS.

When four additional ML methods were used, the best-performing model was the random forest classifier, but XGBoost achieved better performance metrics across the board. Furthermore, a multilayer cross-validation method was used to optimize model hyperparameters and avoid overfitting, thereby boosting model generalizability. Several studies have applied artificial intelligence on oriented radiological tasks, particularly those that differentiate breast cancers as benign or malignant. For example, in 2015, Mandeep Rana et al. [20] developed a Support Vector Machines sequential minimum optimization model that combined a K-nearest neighbors algorithm approach with Manhattan measures and other ML techniques to classify breast cancers. In 2018, Maysanjaya et al. [21] combined two algorithms to develop a Computer-aided Detection based method and a naive Bayes algorithm that achieved an accuracy of 99.27%. Ezgi Mercan et al. [22] built a classification model to discriminate between invasive and non-invasive breast cancer based on breast pathology structures using Digital WSIs for breast biopsies. The accuracy of the model reached 0.98, and the sensitivity was 0.84. In addition, Shikha Roy et al. [23] demonstrated that DCIS and invasive ductal carcinoma can be classified based on gene expression with RNA-seq gene expression profiles from The Cancer Genome Atlas (TCGA). In addition, Niyazi Senturk et al. [24] proposed an AI model to assess the risk of BRCA variation of breast cancer. Unlike the models in these studies, which required the use of many algorithms and a large number of images, ours uses only XGBoost with optimization, reducing training time and model complexity. Furthermore, we focused on distinguishing DCIS from MIBC rather than benign from malignant, a greater challenge for radiologists.

Our model could classify breast cancers with a specificity and sensitivity similar to or greater than those achieved by our radiologists. Greater sensitivities and specificities were achieved by the radiologists when using both MMG and US images compared to using only one of these imaging modalities. This confirms the results of others who have also shown better breast cancer detection when using both MMG and US imaging [25,26]. Compared to a first-year resident, our model’s specificity and sensitivity were greater (0.75 vs. 0.64, *p* < 0.05; and 0.91 vs. 0.74, *p* < 0.05, respectively). Compared to a ten-year veteran radiologist, even with using MMG plus US image, however, the differences were not significant. Even though the ML model cannot replace diagnostics by radiologists, their workloads can be reduced when this ML model is implemented.

This study has several limitations. First, it was implemented in a single center, and external validation of the best-performing model was not performed. Doing so could have further demonstrated its generalizability. Second, it was a retrospective study based on a limited number of patients. Therefore, studies employing larger sample sizes are needed to confirm our results. On the other hand, a deep learning approach, such as a convolutional neural network, might outperform this model when combined with radiomic features (such as MMG image or magnetic resonance images) or genetic data to enhance the performance of our model.

## 5. Conclusions

In conclusion, the XGBoost model developed in this study, when provided with clinical characteristics, mammographic and ultrasonographic findings, and histopathologic features from medical records, can successfully discriminate DCIS from MIBC at the level of experienced radiologists, thereby providing patients with more options for less-invasive therapies.

## Figures and Tables

**Table 1:** Clinical characteristics of the study population.

Characteristic	Study Group (*n* = 420)	DCIS Group (*n* = 189)	MIBC Group (*n* = 231)	*p* Value ^b^
Age ^a^, y	57.1 (12.0)	57.1 (12.0)	57.3 (12.0)	0.694
Age group				0.086
<40 y	22 (5.2)	6 (3.2)	16 (6.8)	…
≥40 y	398 (94.8)	183 (96.8)	215 (93.2)	…
Menopause				0.643
Premenopause	145 (34.5)	63 (33.3)	82 (35.3)	…
Postmenopause	275 (65.5)	126 (66.7)	149 (64.5)	…
Age at menarche				0.837
NA	93 (22.1)	44 (23.2)	49 (21.2)	…
<12 y	27 (6.4)	13 (6.9)	14 (6.1)	…
12–14 y	222 (52.9)	99 (52.4)	123 (53.2)	…
≥15 y	78 (18.6)	33 (17.5)	45 (19.5)	…
Age at first live birth				0.002
<20 y	12 (3.6)	1 (0.7)	11 (6.0)	…
20–29 y	166 (49.7)	91 (60.3)	75 (41.0)	…
≥30 y	82 (24.6)	34 (22.5)	48 (26.2)	…
Nulliparous	74 (22.1)	25 (16.5)	49 (26.8)	…
Family history of BC				0.002
Yes	83 (19.8)	50 (26.5)	33 (14.3)	…
No	333 (80.2)	139 (73.5)	198 (85.7)	…
History of HRT use				0.464
Yes	31 (7.4)	12 (6.3)	19 (8.2)	…
No	389 (92.6)	177 (93.7)	212 (91.8)	…
BMI ^a^ (kg/m²)	24.02 (4.40)	24.02 (4.39)	24.00 (4.41)	0.542
BMI group				0.002
BMI < 18.5 kg/m²	14 (3.33)	4 (2.12)	10 (4.32)	…
18.5 ≤ BMI < 24 kg/m²	227 (54.05)	98 (51.85)	129 (55.84)	…
24 ≤ BMI < 27 kg/m²	82 (19.52)	44 (23.28)	38 (16.45)	…
BMI ≥ 27 kg/m²	97 (23.10)	43 (22.75)	54 (23.39)	…

Unless otherwise indicated, data in the table are expressed as number (percentage) ^a^ Expressed as mean (standard deviation)**.** ^b^ DCIS group vs. MIBC group. DCIS: ductal carcinoma in situ; MIBC: minimally invasive breast cancer; NA: not available; HRT: hormone replacement therapy; BMI: body mass index; BC: breast cancer.

**Table 2:** Characteristics of the training and testing sets.

Characteristic	Training Set	Testing Set	*p* Value
No. of patients	357	63	
DCIS	161 (45.1)	35 (55.5)	
MIBC	196 (54.9)	28 (45.4)	
Age ^a^, y	57.1 (11.6)	58.5 (12.8)	>0.05
BMI ^a^, kg/m^2^	24.1 (4.7)	24.1 (4.9)	>0.05
Premenopause	124 (34.7)	21 (33.3)	>0.05
Postmenopause	233 (65.3)	42 (66.7)	>0.05
Family history of BC	61 (18.7)	16 (25.4)	>0.05

Unless otherwise indicated, data in the table are expressed as number (percentage) ^a^ Expressed as mean (standard deviation). DCIS: ductal carcinoma in situ; MIBC: minimally invasive breast cancer; BMI: body mass index; BC: breast cancer.

**Table 3:** Performance comparisons of five models.

Model	Accuracy	F1 Score	Recall	Precision
XGBoost	0.84 [0.76–0.91]	0.87 [0.79–0.93]	0.91 [0.76–0.94]	0.82 [0.71–0.92]
GaussianNB	0.75 [0.67–0.84]	0.79 [0.67–0.86]	0.88 [0.68–0.93]	0.72 [0.65–0.92]
KNeighbors Classifier	0.63 [0.54–0.69]	0.73 [0.56–0.80]	0.87 [0.57–0.92]	0.62 [0.55–0.90]
DecisionTree Classifier	0.73 [0.64–0.82]	0.76 [0.64–0.84]	0.77 [0.64–0.86]	0.75 [0.64–0.86]
RandomForest Classifier	0.82 [0.74–0.89]	0.84 [0.76–0.91]	0.89 [0.73–0.93]	0.81 [0.78–0.91]

Data in the table are expressed as value [95% confidence interval].

**Table 4:** Performance comparison of the XGBoost model and two radiologists.

	Sensitivity	*p^se^*	Specificity	*p^sp^*
Radiologist 1				
Using MMG alone	0.65 (0.61–0.71)		0.59 (0.57–0.62)	
Using US alone	0.67 (0.62–0.72)		0.59 (0.55–0.63)	
Using both US and MMG	0.74 (0.68–0.79)	<0.05	0.64 (0.57–0.66)	<0.05
Radiologist 2				
Using MMG alone	0.81 (0.74–0.86)		0.68 (0.65–0.72)	
Using US alone	0.77 (0.73–0.82)		0.64(0.61–0.74)	
Using both US and MMG	0.83 (0.74–0.88)	>0.05	0.71 (0.68–0.74)	>0.05
XGBoost Model	0.91 (0.76–0.94)		0.75 (0.68–0.78)	

Data in the table are expressed as value (95% confidence interval)**.** DCIS: ductal carcinoma in situ; MIBC: minimally invasive breast cancer; MMG: mammogram; US: ultrasound. *p^se^*, *p^sp^* indicate the probability of significant differences in sensitivity and specificity, respectively, between the XGBoost model and the radiologist.

## Data Availability

The data that support the findings of this study are available from the corresponding author upon reasonable request.

## Appendix A

Sonographic Features

At our hospital, physicians performed bilateral whole-breast real-time ultrasound imaging on 389 of the 420 patients included in this study. The device used was a high-resolution GE LOGIQ E9 equipped with a high-frequency linear array transducer capable of producing 5 to 13 MHz.

Two radiologists with more than ten years of experience in interpreting breast ultrasound images reviewed the sonographic images, blinded to the pathological results. The sonographic features were interpreted using BI-RADS criteria (5th ed.), analyzing breast composition and the breast mass features of tumor size, shape, orientation, margin, echo pattern, posterior features, calcifications, vascularity, and elasticity assessment. Color power findings using Doppler sonography were routinely reported on hardcopy pictures and in physician notes, indicating the presence or absence of intralesional vascularity. Architectural distortion, ductal changes, and the presence of the axillary lymph node were also noted. Interval changes on follow-up ultrasound were recorded.

Mammographic features

In total, 321 patients underwent mammograms. Standard two-view mammography was performed using a Siemens digital full field system (Mammomat Revelation), and additional views were obtained if necessary. Each set of images was read by two radiologists with more than ten years of experience in the field, applying BI-RADS criteria (5th ed.) to classify the masses (measurements, shape, density, and margin), calcifications (morphology and distribution), and architectural distortion. Asymmetries in density and morphology were noted. Interval changes on follow-up mammograms were also reviewed.

**Table A1:** Description of features.

Features	Description
Age	age of patient
BMI	body mass index of patient
Mass_measure_US	size of mass on ultrasound
Mass_Measure_MMG	size of mass on mammography
Asymetry_measure	size of asymmetry on mammography
Age_N.1	<40
Age_N.2	≥40
Menopause.0	premenopause
Menopause.1	menopause
Menarche.0	not available
Menarche.1	menarche: <12
Menarche.2	menarche: 12–14
Menarche.3	menarche: ≥15
X1st_live_birth.0	age at 1st live birth: <20
X1st_live_birth.1	age at 1st live birth: 20–24
X1st_live_birth.2	age at 1st live birth: 25–29
X1st_live_birth.3	age at 1st live birth: ≥30
X1st_live_birth.4	nulliparous
BMI_n.1	BMI < 18.5
BMI_n.2	BMI 18.5–24
BMI_n.3	BMI 24–27
BMI_n.4	BMI ≥ 27
Family_history.0	have family history of breast cancer
Family_history.1	do not have family history of breast cancer
HRT.0	have history of use hormone replacement therapy
HRT.1	do not have history of use hormone replacement therapy
Chief_complaint.0	screening
Chief_complaint.1	have symptom
Breast_composition_US.1	breast composition category A on ultrasound
Breast_composition_US.2	breast composition category B on ultrasound
Breast_composition_US.3	breast composition category C on ultrasound
Mass_US.0	have mass in ultrasound
Mass_US.1	do not have mass in ultrasound
Mass_shape_US.0	not available
Mass_shape_US.1	oval-shape
Mass_shape_US.2	round-shape
Mass_shape_US.3	irregular-shape
Mass_Orientation_US.0	not available
Mass_Orientation_US.1	parallel
Mass_Orientation_US.2	not parallel
Mass_Margin_US.0	not available
Mass_Margin_US.1	circumscribed
Mass_Margin_US.2	indistinct
Mass_Margin_US.3	angular
Mass_Margin_US.4	microlobulated
Mass_Margin_US.5	spiculated
Mass_Echopattern_US.0	not available
Mass_Echopattern_US.1	anechoic
Mass_Echopattern_US.2	hyperechoic
Mass_Echopattern_US.3	complex cystic and solid
Mass_Echopattern_US.4	hyperechoic
Mass_Echopattern_US.5	isoechoic
Mass_Echopattern_US.6	heterogenous
Mass_Posterior_features.0	not available
Mass_Posterior_features.1	no posterior features
Mass_Posterior_features.2	enhancement
Mass_Posterior_features.3	shadowing
Mass_Posterior_features.4	combined pattern
Mass_Vascularity.0	not available
Mass_Vascularity.1	absent
Mass_Vascularity.2	internal vascularity
Mass_Vascularity.3	vessels in rim
Mass_Elasticity_assessment.0	not available
Mass_Elasticity_assessment.1	soft
Mass_Elasticity_assessment.2	intermediate
Mass_Elasticity_assessment.3	hard
Calcifications_US.1	calcifications in a mass
Calcifications_US.2	calcifications outside of a mass
Calcifications_US.3	intraductal calcifications
Calcifications_US.4	no calcifications
Architectural_distortion_US.1	have architectural distortion
Architectural_distortion_US.2	do not have architectural distortion
Duct_changes.1	not available
Duct_changes.2	ectasia
Duct_changes.3	dilation
Duct_changes.4	calcification
Lymph_nodes_US.0	do not have lymph nodes
Lymph_nodes_US.1	have lymph nodes
Interval_change_US.0	do not have interval change on ultrasound
Interval_change_US.1	have interval change on ultrasound
Interval_change_US.2	no previous ultrasound
BIRAD_US.0	not available
BIRAD_US.1	BIRADS ultrasound 0
BIRAD_US.2	BIRADS ultrasound 1
BIRAD_US.3	BIRADS ultrasound 2
BIRAD_US.4	BIRADS ultrasound 3
BIRAD_US.5	BIRADS ultrasound 4
BIRAD_US.6	BIRADS ultrasound 5
Breast_composition_MMG.1	breast composition category A on mammography
Breast_composition_MMG.2	breast composition category B on mammography
Breast_composition_MMG.3	breast composition category C on mammography
Breast_composition_MMG.4	breast composition category D on mammography
Mass_MMG.0	do not have mass in mammography
Mass_MMG.1	have mass in mammography
Mass_shape.0	not available
Mass_shape.1	oval-shape on mammography
Mass_shape.2	round-shape on mammography
Mass_shape.3	irregular-shape on mammography
Mass_margin.0	not available
Mass_margin.1	circumscribed mass on mammography
Mass_margin.2	obscured mass on mammography
Mass_margin.3	microlobulated mass on mammography
Mass_margin.4	indistinct mass on mammography
Mass_margin.5	spiculated mass on mammography
Mass_density.0	not available
Mass_density.1	high density
Mass_density.2	equal density
Mass_density.3	low density
Mass_density.4	fat-containing
Calcification_MMG_1	have suspicious morphology calcification on mammography
Calcification_MMG_0	do not have suspicious morphology calcification on mammography
Sus_Amorphous.1	amorphous calcifications on mammography
Sus_Amorphous.0	do not have amorphous calcifications on mammography
Sus_Coarseheterogeneous.1	coarse heterogeneous calcifications on mammography
Sus_Coarseheterogeneous.0	do not have coarse heterogenous calcifications on mammography
Sus_Finepleomorphic.1	fine pleomorphic calcifications on mammography
Sus_Finepleomorphic.0	do not have fine pleomorphic calcifications on mammography
Sus_Finelinear_branching.1	fine linear or fine linear branching calcifications on mammography
Sus_Finelinear_branching.0	do not have fine linear or fine linear branching calcifications on mammography
Sus_Diffuse.1	distribution diffuse suspicious calcification on mammography
Sus_Diffuse.0	do not have distribution diffuse suspicious calcification on mammography
Sus_Regional.1	distribution regional suspicious calcification on mammography
Sus_Regional.0	do not have distribution regional suspicious calcification on mammography
Sus_Grouped.1	distribution grouped suspicious calcification on mammography
Sus_Grouped.0	do not have distribution grouped suspicious calcification on mammography
Sus_Linear.1	distribution linear suspicious calcification on mammography
Sus_Linear.0	do not have distribution linear suspicious calcification on mammography
Sus_Segmental.1	distribution segmental suspicious calcification on mammography
Sus_Segmental.0	do not have distribution segmental suspicious calcification on mammography
Asymetries.0	asymmetry
Asymetries.1	global asymmetry
Asymetries.3	focal asymmetry
Asymetries.4	developing asymmetry
Asfeatures_Nippleretraction.0	do not have associated features: nipple retraction
Asfeatures_Nippleretraction.1	associated features: nipple retraction
Asfeatures_Skinthickening.0	do not have associated features: skin thickening
Asfeatures_Skinthickening.1	associated features: skin thickening
Asfeatures_Axillaryadenopathy.0	do not have associated features: axillary adenopathy
Asfeatures_Axillaryadenopathy.1	associated features: axillary adenopathy
Asfeatures_tissue_retraction.0	do not have associated features: tissue retraction
Asfeatures_tissue_retraction.1	associated features: tissue retraction
Interval_change_MMG.0	do not have interval change on mammography
Interval_change_MMG.1	have interval change on mammography
Interval_change_MMG.2	no previous mammography
BIRADS_MMG.0	not available
BIRADS_MMG.1	BIRADS MMG 0
BIRADS_MMG.2	BIRADS MMG 1
BIRADS_MMG.3	BIRADS MMG 2
BIRADS_MMG.4	BIRADS MMG 3
BIRADS_MMG.5	BIRADS MMG 4A
BIRADS_MMG.6	BIRADS MMG 4B
BIRADS_MMG.7	BIRADS MMG 4C
BIRADS_MMG.8	BIRADS MMG 5
ER.0	ER negative
ER.1	ER positive
PR.0	PR negative
PR.1	PR positive
Her2.0	HER2 negative
Her2.1	HER2 positive
Cribriform.0	do not have architectural pattern: cribriform
Cribriform.1	have architectural patterns: cribriform
Papillary.0	do not have architectural pattern: papillary
Papillary.1	have architectural pattern: papillary
Solid.0	do not have architectural pattern: solid
Solid.1	have architectural pattern: solid
Comedo.0	do not have architectural pattern: comedo
Comedo.1	have architectural pattern: comedo
Micropapillary.0	do not have architectural pattern: micropapillary
Micropapillary.1	have architectural pattern: micropapillary
Paget.0	do not have architectural pattern: paget
Paget.1	have architectural pattern: paget
Flat.0	do not have architectural pattern: flat (clinging)
Flat.1	have architectural pattern: flat (clinging)
Miccrocal_in_DCIS.0	do not have microcalcification on pathology pattern
Miccrocal_in_DCIS.1	have microcalcification on pathology pattern
Miccrocal_in_nonneoplastic.0	do not have microcalcification in non- neoplastic tissue
Miccrocal_in_nonneoplastic.1	have microcalcification in non- neoplastic tissue
Miccrocal_in_invasive_carcinoma.0	do not have microcalcification in invasive carcinoma
Miccrocal_in_invasive_carcinoma.1	have microcalcification in invasive carcinoma
Necrosis.0	necrosis not available
Necrosis.1	necrosis in focal (small foci or single cell necrosis)
Necrosis.2	necrosis in central (expansive comedo necrosis)
Nuclear_grade.1	nuclear grade i
Nuclear_grade.2	nuclear grade ii
Nuclear_grade.3	nuclear grade iii
Palpable_lump.0	do not have palpable lump
Palpable_lump.1	have palpable lump

## Appendix B

**Table A2:** Hyperparameter tuning of XGBoost.

Parameters	Index
learning_rate	0.03
gamma	0
max_depth	6
colsample_bylevel	0.06
colsample_bytree	0.61
colsample_bynode	1
subsample	0.7
n_estimators	200

**Table A3:** Feature contribution by XGBoost.

Feature	XGBoost_Importance _Score
Calcification_MMG_1	0.860544218
Mass_Echopattern_US.4	0.795918367
Mass_Orientation_US.2	0.765306122
Mass_Posterior_features.4	0.765306122
Lymph_nodes_US.1	0.741496599
Calcification_MMG_0	0.741496599
BMI	0.707482993
PR.1	0.704081633
Miccrocal_in_invasive_carcinoma.0	0.704081633
Mass_Posterior_features.2	0.697278912
Mass_Posterior_features.0	0.693877551
ER.1	0.676870748
ER.0	0.676870748
Age	0.659863946
Mass_Vascularity.0	0.656462585
Mass_Vascularity.2	0.653061224
Sus_Grouped.1	0.649659864
Sus_Grouped.0	0.649659864
Mass_Measure_US	0.629251701
Mass_US.0	0.62585034
Mass_Echopattern_US.0	0.62585034
Mass_US.1	0.62585034
Sus_Coarseheterogeneous.1	0.62585034
Sus_Coarseheterogeneous.0	0.62585034
Papillary.0	0.62244898
Mass_Measure_MMG	0.62244898
Sus_Amorphous.0	0.62244898
Sus_Amorphous.1	0.62244898
Papillary.1	0.62244898
Sus_Finepleomorphic.1	0.619047619
Sus_Finepleomorphic.0	0.619047619
Mass_Posterior_features.1	0.615646259
Mass_Vascularity.1	0.612244898
Solid.0	0.612244898
Solid.1	0.612244898
Sus_Linear.0	0.605442177
Sus_Linear.1	0.605442177
Mass_MMG.1	0.605442177
Mass_MMG.0	0.605442177
Breast_composition_MMG.3	0.605442177
BIRAD_US.0	0.602040816
Sus_Regional.1	0.595238095
Sus_Regional.0	0.595238095
Mass_shape_US.0	0.591836735
Sus_Segmental.0	0.588435374
Sus_Segmental.1	0.588435374
Mass_shape_US.1	0.588435374
Sus_Finelinear_branching.1	0.588435374
Mass_Margin_US.0	0.588435374
Sus_Finelinear_branching.0	0.588435374
Mass_Posterior_features.3	0.585034014
Mass_margin.0	0.585034014
Nuclear_grade.1	0.581632653
Cribriform.0	0.581632653
Cribriform.1	0.581632653
Breast_composition_MMG.1	0.581632653
Nuclear_grade.2	0.581632653
Mass_density.1	0.578231293
Mass_shape_US.3	0.578231293
Mass_density.0	0.578231293
BMI_n.3	0.578231293
Family_history.1	0.574829932
Family_history.0	0.574829932
Micropapillary.0	0.571428571
Duct_changes.4	0.571428571
Micropapillary.1	0.571428571
BIRADS_MMG.1	0.568027211
Duct_changes.1	0.568027211
Duct_changes.3	0.56462585
Mass_shape.3	0.56462585
Chief_complaint.0	0.56462585
Menarche.1	0.56122449
Asymmetry_measure	0.56122449
Miccrocal_in_nonneoplastic.0	0.557823129
Miccrocal_in_DCIS.1	0.557823129
PR.0	0.557823129
Miccrocal_in_nonneoplastic.1	0.557823129
Miccrocal_in_invasive_carcinoma.0	0.557823129
Miccrocal_in_invasive_carcinoma.1	0.557823129
Palpable_lump.0	0.557823129
Her2.1	0.557823129
Asymetries.4	0.557823129
Her2.0	0.557823129
Asymetries.0	0.557823129
BIRADS_MMG.5	0.557823129
Paget.0	0.557823129
Comedo.1	0.557823129
Paget.1	0.557823129
BIRADS_MMG.4	0.557823129
BIRADS_MMG.3	0.557823129
BIRADS_MMG.0	0.557823129
Interval_change_MMG.1	0.557823129
Interval_change_MMG.0	0.557823129
Asfeatures_tissue_retraction.1	0.557823129
Asfeatures_tissue_retraction.0	0.557823129
Asfeatures_.Axillaryadenopathy.1	0.557823129
Asfeatures_.Axillaryadenopathy.0	0.557823129
Asfeatures_.Skinthickening.1	0.557823129
Flat.1	0.557823129
Asfeatures_.Skinthickening.0	0.557823129
Asfeatures_Nippleretraction.1	0.557823129
Comedo.0	0.557823129
Asfeatures_Nippleretraction.0	0.557823129
Asymetries.3	0.557823129
Asymetries.1	0.557823129
Flat.0	0.557823129
Breast_composition_MMG.4	0.557823129
Sus_Diffuse.1	0.557823129
Sus_Diffuse.0	0.557823129
Mass_margin.3	0.557823129
Mass_margin.1	0.557823129
Lymph_nodes_US.1	0.557823129
Mass_Orientation_US.1	0.557823129
Mass_shape.1	0.557823129
Breast_composition_US.3	0.557823129
Breast_composition_US.2	0.557823129
Breast_composition_US.1	0.557823129
Chief_complaint.1	0.557823129
Chief_complaint.0	0.557823129
HRT.1	0.557823129
HRT.0	0.557823129
BMI_n.4	0.557823129
BMI_n.1	0.557823129
BMI_n.2	0.557823129
X1st_live_birth.4	0.557823129
X1st_live_birth.3	0.557823129
X1st_live_birth.1	0.557823129
X1st_live_birth.0	0.557823129
Menarche.3	0.557823129
Menarche.0	0.557823129
Menopause.1	0.557823129
Menopause.0	0.557823129
Age_N.2	0.557823129
Age_N.1	0.557823129
Mass_margin.4	0.557823129
Mass_margin.5	0.557823129
Mass_Echopattern_US.1	0.557823129
Interval_change_US.0	0.557823129
Mass_margin.1	0.557823129
Mass_margin.3	0.557823129
Mass_margin.2	0.557823129
Mass_density.4	0.557823129
Mass_shape.1	0.557823129
Breast_composition_MMG.1	0.557823129
BIRAD_US.5	0.557823129
BIRAD_US.4	0.557823129
BIRAD_US.3	0.557823129

**Table A4:** Feature contribution by SHAP.

Feature	SHAP_Importance_Score
Calcification_MMG_1	1.530532241
Mass_Orientation_US.2	0.843982518
Miccrocal_in_invasive_carcinoma.0	0.394437432
Mass_Posterior_features.3	0.278461546
BMI_n.2	0.272588193
Age	0.240817562
Papillary.0	0.188818902
X1st_live_birth.2	0.182896554
Mass_measure_US	0.159969047
Solid.0	0.120786794
Nuclear_grade.2	0.097087704
Breast_composition_US.3	0.088376589
Mass_Elasticity_assessment.0	0.085524194
Family_history.0	0.080155298
Mass_shape_US.3	0.07565444
Calcifications_US.2	0.066437013
Her2.0	0.062137935
Mass_Vascularity.2	0.062031701
BIRADS_MMG.5	0.058612607
BMI_n.4	0.058029428

## References

[1] Sung H., Ferlay J., Siegel R.L., Laversanne M., Soerjomataram I., Jemal A., Bray F. (2021). Global cancer statistics 2020: GLOBOCAN estimates of incidence and mortality worldwide for 36 cancers in 185 countries. CA Cancer J. Clin..

[2] Kerlikowske K. (2010). Epidemiology of ductal carcinoma in situ. J. Natl. Cancer Inst. Monogr..

[3] Lee R.J., Vallow L.A., McLaughlin S.A., Tzou K.S., Hines S.L., Peterson J.L. (2012). Ductal carcinoma in situ of the breast. Int. J. Surg. Oncol..

[4] Alkabban F., Ferguson T. (2021). Breast Cancer.

[5] Sharma G.N., Dave R., Sanadya J., Sharma P., Sharma K. (2010). Various types and management of breast cancer: An overview. J. Adv. Pharm. Technol. Res..

[6] Tabár L., Chen H.-H., Duffy S.W., Yen M., Chiang C., Dean P.B., Smith R.A. (2000). A novel method for prediction of long-term outcome of women with T1a, T1b, and 10–14 mm invasive breast cancers: A prospective study. Lancet.

[7] Tabar L., Tony Chen H.H., Amy Yen M., Tot T., Tung T.H., Chen L.S., Chiu Y.H., Duffy S.W., Smith R.A. (2004). Mammographic tumor features can predict long-term outcomes reliably in women with 1–14-mm invasive breast carcinoma: Suggestions for the reconsideration of current therapeutic practice and the TNM classification system. Cancer Interdiscip. Int. J. Am. Cancer Soc..

[8] Silverstein M.J., Lagios M.D., Craig P.H., Waisman J.R., Lewinsky B.S., Colburn W.J., Poller D.N. (1996). A prognostic index for ductal carcinoma in situ of the breast. Cancer Interdiscip. Int. J. Am. Cancer Soc..

[9] Tabár L., Duffy S.W., Vitak B., Chen H.H., Prevost T.C. (1999). The natural history of breast carcinoma: What have we learned from screening?. Cancer.

[10] Joensuu H., Pylkkänen L., Toikkanen S. (1999). Late mortality from pT1N0M0 breast carcinoma. Cancer.

[11] Koscielny M.T. (1999). Serge The rationale for early diagnosis of cancer: The example of breast cancer. Procedia Comput. Sci..

[12] Vaka A.R., Soni B., Reddy S. (2020). Breast cancer detection by leveraging Machine Learning. ICT Express.

[13] Naji M.A., El Filali S., Aarika K., Benlahmar E.H., Abdelouhahid R.A., Debauche O. (2021). Machine Learning Algorithms For Breast Cancer Prediction And Diagnosis. Procedia Comput. Sci..

[14] D’Orsi C.J., Sickles E.A., Mendelson E.B., Morris E.A., Bassett L.W., Böhm-Vélez M., Comstock C.E., CH L. (2014). ACR BI-RADS® Atlas, Breast Imaging Reporting and Data System.

[15] Brinton L.A., Hoover R., Fraumeni J.F. (1983). Epidemiology of minimal breast cancer. JAMA.

[16] Chen T., Guestrin C. XGBoost: A Scalable Tree Boosting System. Proceedings of the The 22nd ACM SIGKDD International Conference.

[17] Lundberg S.M., Lee S.-I. A unified approach to interpreting model predictions. Proceedings of the Advances in Neural Information Processing Systems 30 (NIPS 2017).

[18] Kim S.H., Seo B.K., Lee J., Kim S.J., Cho K.R., Lee K.Y., Je B.-K., Kim H.Y., Kim Y.-S., Lee J.-H. (2008). Correlation of ultrasound findings with histology, tumor grade, and biological markers in breast cancer. Acta Oncol..

[19] Chen S., Cheung Y., Lo Y., Chen M., Hwang T., Su C., Hsueh S. (2003). Sonographic differentiation of invasive and intraductal carcinomas of the breast. Br. J. Radiol..

[20] Rana M., Chandorkar P., Dsouza A., Kazi N. (2015). Breast cancer diagnosis and recurrence prediction using machine learning techniques. Int. J. Res. Eng..

[21] Maysanjaya I., Pradnyana I., Putrama I. (2018). Classification of breast cancer using Wrapper and Naïve Bayes algorithms. J. Phys. Conf. Ser..

[22] Mercan E., Mehta S., Bartlett J., Shapiro L.G., Weaver D.L., Elmore J.G. (2019). Assessment of Machine Learning of Breast Pathology Structures for Automated Differentiation of Breast Cancer and High-Risk Proliferative Lesions. JAMA Netw. Open.

[23] Roy S., Kumar R., Mittal V., Gupta D. (2020). Classification models for Invasive Ductal Carcinoma Progression, based on gene expression data-trained supervised machine learning. Sci. Rep..

[24] Senturk N., Tuncel G., Dogan B., Aliyeva L., Dundar M.S., Ozemri Sag S., Mocan G., Temel S.G., Dundar M., Ergoren M.C. (2021). BRCA Variations Risk Assessment in Breast Cancers Using Different Artificial Intelligence Models. Genes.

[25] Rebolj M., Assi V., Brentnall A., Parmar D., Duffy S.W. (2018). Addition of ultrasound to mammography in the case of dense breast tissue: Systematic review and meta-analysis. Br. J. Cancer.

[26] Berg W.A., Blume J.D., Cormack J.B., Mendelson E.B., Lehrer D., Böhm-Vélez M., Pisano E.D., Jong R.A., Evans W.P., Morton M.J. (2008). Combined screening with ultrasound and mammography vs mammography alone in women at elevated risk of breast cancer. JAMA.

